# Online Prediction of Molded Part Quality in the Injection Molding Process Using High-Resolution Time Series

**DOI:** 10.3390/polym15040978

**Published:** 2023-02-16

**Authors:** Lucas Bogedale, Stephan Doerfel, Alexander Schrodt, Hans-Peter Heim

**Affiliations:** 1 Faculty of Mechanical Engineering, Institute of Materials Engineering-Plastics, University of Kassel, 34125 Kassel, Germany; 2 Faculty of Computer Science and Electrical Engineering, Data Science, Kiel University of Applied Sciences, 24149 Kiel, Germany; 3 Micromata GmbH, 34131 Kassel, Germany; 4 Institute of Physics, Functional Thin Films and Physics with Syncrotron Radiation, University of Kassel, 34127 Kassel, Germany; 5 Data Hive Cassel GmbH, 34130 Kassel, Germany

**Keywords:** injection molding, process monitoring, online part quality prediction, time series

## Abstract

Process-data-supported process monitoring in injection molding plays an important role in compensating for disturbances in the process. Until now, scalar process data from machine controls have been used to predict part quality. In this paper, we investigated the feasibility of incorporating time series of sensor measurements directly as features for machine learning models, as a suitable method of improving the online prediction of part quality. We present a comparison of several state-of-the-art algorithms, using extensive and realistic data sets. Our comparison demonstrates that time series data allow significantly better predictions of part quality than scalar data alone. In future studies, and in production-use cases, such time series should be taken into account in online quality prediction for injection molding.

## 1. Introduction

Injection molding is one of the most widely used industrial plastic processing methods. Around 110,000 new injection molding machines are put into operation worldwide every year. With an average service life of 10 years, more than 1 million injection molding machines are currently in industrial use. On average, about five to six molds run on each of these machines. As a specific configuration is set for each mold, 5 to 6 million different running injection molding processes can be expected [1].

Despite extensive setting parameters and integrated control systems in standard injection molding machines, the influence of disturbance variables repeatedly causes fluctuations in process stability during ongoing production, and thus, deviations in the quality of the injection-molded parts.

To compensate for the resulting production capacity losses, many approaches to monitoring and optimizing the injection molding process have been developed. Recent approaches have been dedicated to the evaluation of process data, which provide information about the course of the injection molding process: based on this data, disturbing influences on the process can be detected at an early stage, and dampened or compensated for accordingly. The process data come from pressure, temperature, force, and displacement sensors, and are collected during each injection molding cycle. Many studies have shown how partial aspects of the injection molding process can be monitored or optimized based on indices derived from the process data, or modeling concepts based on process data [2,3,4,5,6,7,8].

One approach to detecting process instabilities in the injection molding process is to predict the quality properties of the molded parts. Consistency in the part quality properties of the molded parts is crucial for every processor. Predicted quality properties can be used similarly to measured ones, e.g., for the evaluation of process capability; however, permanent automatic online measurement of gravimetric and geometrical quality properties on every injection molding machine is not an option for most industrial applications, due to the high costs of such measurements (manual work or dedicated additional hardware). In contrast to such part quality data measurements, process data from a machine’s internal sensors are already provided within standard injection molding machine controls. The main purpose of the sensors is internal process control: if the sensory process data can be made available, it can also serve additional purposes, e.g., the predicting of molding quality without explicitly measuring it on the product. Recent work has shown that part quality prediction is possible using machine learning (ML) algorithms: in this context, the features that serve as input for the algorithms are measurements represented as scalar values, i.e., single numerical aggregates, such as maximum injection pressure, residual mass cushion, and metering time [9].

In this paper, we investigated the use of full time series of sensor measurements in place of, or in addition to, such scalar features. Time series can be continuously collected from a machine’s internal sensors throughout each injection molding cycle. A time series usually includes injection pressure over time, injection flow over time, and cavity pressure over time. Recent work has shown that scalar features derived from time series are relevant, in the context of part quality prediction, and can lead to an improvement in prediction accuracy. Ke et al. used indices (integrals and maxima) extracted from cavity pressure and injection pressure curves, and achieved promising prediction accuracy for geometric dimensions, using a multilayer perceptron mesh model [10]. Huang et al. extended this approach, by integrating an autoencoder network for automated feature extraction [11]. Párizs et al. demonstrated another approach, whereby features were generated from cavity pressure curves, by forming integrals for quality prediction in a multi-cavity injection molding process [12].

Scalar values, such as the maximum injection pressure or the switchover injection pressure, represent only one value at a specific point in time during the molding process. Scalar indices, which are derived from time series, extract specific information, by discarding other information from the data set: by contrast, high-resolution time series contain a large number of individual values, and thus more information about the course of a cycle. The high information content of the time series can lead to inaccurate and biased models, due to overfitting, in classical modeling approaches, but is particularly well-suited to the use of machine learning methods, if the right precautions are taken. Very few studies have been conducted using complete time series directly as features—i.e., without aggregating the series into scalar features—for ML models in quality prediction: Nagorny et al. utilized Long Short-Term Memory networks on a small data set (204 samples), to make quality predictions from time series data, but required in-mold pressure and in-mold temperature sensors [13]. Chen et al. obtained promising results, using time series with self-organizing maps and a back-propagation neural network model based on one data set with 180 samples [14]. Both studies showed the benefits of using non-aggregated time series, including data from in-mold sensors. Thus, at this stage it is unclear how generalizable and reliable such results are, or if the inclusion of time series is also beneficial using only data exported from unmodified standard injection molding machines, without the need for in-mold sensor data.

Learning ML algorithms are known to depend on random influences, such as the splitting of the data into test and training sets, the initialization of parameters, or random choices in the learning steps. Moreover, to assess generalization, it is imperative to test the inclusion of time series data on diverse, large-scale data sets: therefore, in this article, we approached this task by conducting extensive experiments on three extensive data sets, using a resilient ML setup and multiple algorithms relying on different prediction principles to get reliable results.

For the successful application of online part quality prediction in industrial applications, the accuracy of the predictions—the deviation between predicted and actual value for a specific quality property—must be reliably high. As the relationships between process data and part quality are very complex, machine learning models are employed to automatically learn functions that approximate the actual quality property based on certain sensor data features: their accuracy depends on the choice of features (the type of process data), and on an appropriate mathematical approximation model (the machine learning method). For a reliable impression of the performance of such models and the selected features, it is imperative to test and evaluate them on the basis of extensive data sets that represent a realistic, industrial injection molding process. As mentioned above, the learning process in ML involves random choices: therefore, such approaches have to be compared in repeated experiments, to mitigate the influence of random factors.

To that end, this paper examined and compared several state-of-the-art machine learning algorithms, to assess their prediction performance. To determine the influence of the feature data category on the prediction quality, each combination of model and feature data category was evaluated separately: this meant that, initially, all machine learning models were trained solely on scalar data, as in previous approaches; then, similar evaluations were conducted, but using either the time series only or the combination of both scalar and time series data.

For the experiments, we created three extensive data sets of cycles and parts, containing 1167 samples, 829 samples, and 1332 samples, respectively: thus, we were able to compare the algorithms’ performance for different types of produced parts, made of polyamide filled with 30% glass fiber, from two different manufacturers (see Section 2.2). Moreover, in each data set, various process states were artificially induced by manually changing the disturbance variables: thus, the data sets were more representative of various actual industrial production environments and, therefore, the resulting machine learning models were more broadly applicable.

Each data set contained scalar quantities as well as time series; furthermore, for each produced part, two quality properties were recorded: the weight and a geometric dimension of the molded part. It is the goal of this research to make such quality measurements gratuitous in the industrial setting; however, for the experiments, these measurements were used as a gold standard against which the machine learning model predictions were compared.

In the selection of the sensors whose data measurements were included in the study, again we focused on broad applicability: industrially used injection molds are not always equipped with in-mold sensors, for technical or economic reasons, while sensor data for injection pressure and injection flow curves can be made available on all modern injection molding machines; therefore, such sensors were deliberately omitted in this study, in order to make the findings applicable to molds without them.

The three data sets were collected through a novel software, called AVAPS, that allows the real-time query of high-resolution time series in addition to scalar data from standard (not modified for research purposes) injection moldings machines, under industrial conditions: this approach allowed us to export high-resolution (>100 Hz) time series inline from a standard injection molding machine control, without the use of additional hardware, such as measurement amplifiers. AVAPS directly provides the means to run the most successful models in industry, without further hardware or any kind of modification to the machines.

In summary, the three main contributions of this article are:

1. Through a novel comparison, it is shown that high-resolution time series fed directly into ML models—without reducing their information content through the prior formation of indices—are essential features for quality prediction. This is shown by comparing models using only scalar data, only time series data, and a combination of both, as features in state-of-the-art machine learning models.
2. The presented approaches are feasible for the quality prediction of part weights and geometric dimensions, and achieve high prediction quality only on the basis of the high-resolution injection pressure and injection flow curves from the machine. All the data used are available on modern standard injection molding machines, without the need for in-mold sensors or other additional hardware.
3. Large-volume experiments were carried out, in which realistic manufacturing conditions were simulated by artificially inducing disturbances. The resulting extensive data sets allowed the validation of the findings, suggesting their generalizability for similar injection molding processes: thus, they can serve as a baseline for future research. The data will be made publicly available with the publication of this study.

## 2. Materials and Methods

In the following, Section 2.1 describes the equipment and experimental setup used to generate the data. The general structure of the data sets is explained in Section 2.2. In Section 2.2.2, Section 2.2.3 and Section 2.2.4, the individual data sets are presented, the underlying experimental plans are explained, and the measured part properties are shown.

### 2.1. Experimental Setup

All experiments were carried out in a specially built, fully automatic injection molding measuring cell. The aim was to operate an unmodified injection molding machine in industrial, fully automatic mode, while retrieving, in real time, all process data from the machine control and, at the same time, to be able to measure the part quality characteristics online, without human influence and without varying time delays, over several hundred injection molding cycles.

A conventional injection molding machine Allrounder 520E 1500-800 (manufactured by Arburg GmbH + Co KG, 72290 Loßburg, Germany), with a screw diameter of 45 mm, was used. Two different single-cavity molds, with a hot runner, were mounted on the machine, for the experiments. Both the molds and the part geometries were chosen to represent an industrial application in the experiments. The injection molding machine was equipped with an OPC UA server interface, according to the EUROMAP 63 standard. The machine was integrated into a local IP network. A standalone software tool (AVAPS 1.0, 34131 Kassel, Germany) was programmed to retrieve the static data and time series from the OPC UA interface of the machine control. AVAPS enables the querying of all data from the machine control, which are offered under so-called node IDs in the machine-internal OPC UA server. The data are then stored in a specialized database, and can be exported in suitable data formats or passed internally to a machine learning model for evaluation. All data queries and collections can be performed over any number of cycles, during a fully automated industrial injection molding process. The assignment of the individual data—both time series and static data–to one other and to the respective cycle or molded part, is ensured. The software tool is run on a conventional personal computer or server, and connected to the machine via the IP network. The controller of the machine is configured to provide time series with a sampling time of 6 ms. Following the results given in [15], sample rates of 100 Hz and more lead to very good results for machine learning models part quality prediction. With slower sampling, the results deteriorated in the studied example: for this reason, sampling rates of 100 Hz or more are referred to as high sampling rates in the following, and the sampling time of 6 ms (166 Hz) used in this study was thus within this range.

In order to collect the geometric dimensions of the components, a digital measurement projector was added to the injection molding measurement cell. The measurement projector was a IM-7020 (manufactured by Keyence Cooporation 1-3-14, Higashinakajima, Hihashiyodogawa-ku, Osaka, 533-8555, Japan), which enables optical (contactless) 2D measurement of the geometry of the molded parts. The projector had a maximum measurement deviation of 8 μm. The measurement projector was integrated into the test setup. At each cycle, the handling robot took the molded part out of the mold, and placed it on the object table of the measurement projector. The measurement was triggered automatically by a signal from the robot. After the measurement, the molded part was picked up again by the robot, and fed to a scale.

The scale was an Entris BCE323i-1S precision balance from Sartorius, with a maximum linearity deviation of 2 mg. After gravimetric measurement, the next part was fed to the quality measurement setup. The whole process took place within the cycle time of the injection molding machine. All data—both process data and quality data—were stored together, and assigned to the respective machine cycle in the AVAPS database. Note that the setup—with a robot, measurement projector, and scale—was only added to create extensive quality data that could be used to initially train the model and evaluate against the respective predictions from the machine learning algorithms. When using the quality prognosis on an injection molding process in a real industrial application, this part of the setup can be omitted (provided that the quality predictions are precise enough): predicting part quality with a successfully trained model only requires querying the process data via OPC UA from the injection molding machine control, and processing the data in the trained model.

### 2.2. Data Sets

The main concerns in the evaluation of machine learning models are their ability to generalize (i.e., to be applicable in various different situations), and their dependence on the experimental conditions in which they were learned: therefore, we evaluated the same models on three different data sets that were created under different conditions. For each data set, we recorded a large number of cycles over several days, thus simulating naturally occurring process influences. Additionally, we artificially induced process influences—different setting parameter sets and process influencing factors, as they occurred under realistic process conditions—which led to diverse and therefore more representative data sets.

This approach made it possible to represent, as comprehensively as possible, most of the process states in the data sets that can occur in an industrial injection molding process over several thousand cycles over a long period of time. Training machine learning algorithms on such data tackled the problem of having to perform extensive recordings of such data on “real” industrial processes, which is very demanding due to the high time and technical requirements for 100% measurement of the molded part quality.

Furthermore, to examine the behavior of machine learning algorithms in different processes, two molds with completely different process settings were used, as well as two types of polyamide granules.

#### 2.2.1. Data Set General Structure

The data sets consisted of the recorded process data from the injection molding machine control, and the measurement data from the quality measuring devices. Both data categories were assigned to the respective machine cycle counter while the experiments were carried out, and were therefore unmistakably assigned to each other and to the respective injection molding cycle and molded part. The process data could again be divided into two categories: on the one hand, they consisted of the scalar data that can usually also be seen in the actual value log of an injection molding machine control (max. injection pressure, switchover injection pressure, melt cushion, injection time, hot runner temperature, and cylinder heating zone temperatures 2–8); on the other hand, they consisted of time series, which were the injection pressure curve and the injection flow curve that were recorded during each cycle. Each of the time series had a high-resolution sample rate of 6 ms, and consisted of 2049 data points. The measurement data consisted of the scalar values from the scale (part weight) and the digital measurement projector (geometric measurements). Fifteen different geometric measurements were collected from each of the two parts. For this study, the measure with the largest variance was used for the evaluation. The selection of geometric measurements is described below specifically for each data set. In the following, the process data are referred to as features, and the quality measurement data as targets.

#### 2.2.2. Housing Part Data Set

**Figure 1:**
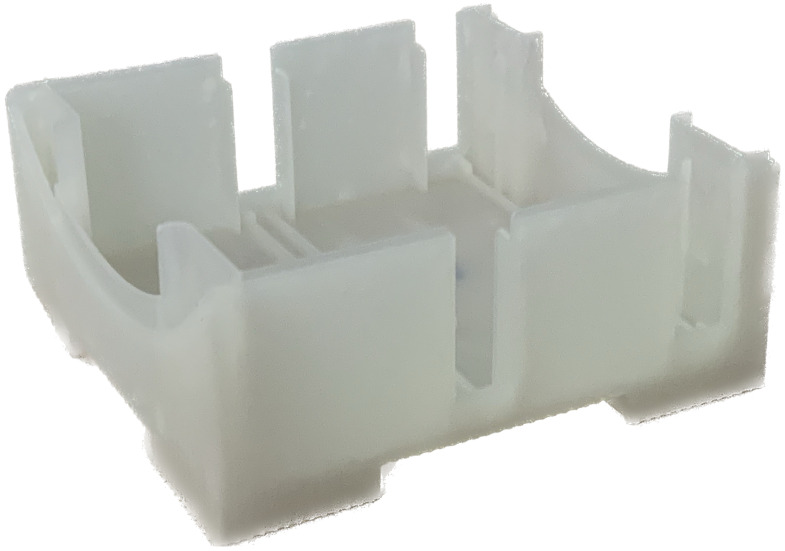
Housing part.

For the experiments to generate the first data set, a mold for a housing part with external dimensions of about 99 × 90 × 42 mm (length × width × height) and a part weight of around 59 g (PA6 30GF) was used (Figure 1). The processed plastic material (Dinalon® B1S25 G30-0288, manufactured by Repol S.L., 12550 Almazora Castellón, Spain) was dried according to the manufacturer’s specifications. The housing part consisted of a complex structure and features that were thin (0.5 mm), partially double-walled and ribbed structures. The process conditions were changed in several trials (Table 1) over four days. At the beginning of each test day, several cycles were performed without changing any parameters: this allowed typical start-up states of the machine, up to the stable running states, to be represented in the data. After that, the barrel and hot runner temperatures, the mold temperatures, and the injection flow were varied at different levels. In addition, pause times typical in practice were induced: for this reason, the machine was stopped at the beginning of the respective stage, for the period of the specified pause times. The melt remained in the heated cylinder during this time, and there was no exchange of the melt after the pausing time had elapsed. The machine was then operated in fully automatic mode. The mold temperature was varied on four levels.

**Figure 2:**
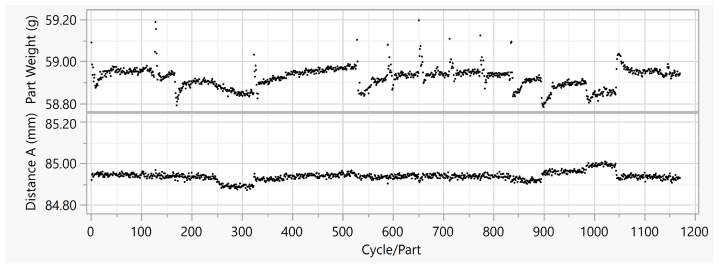
Part weight and Distance A vs. cycle or part in Housing Part data set.

**Figure 3:**
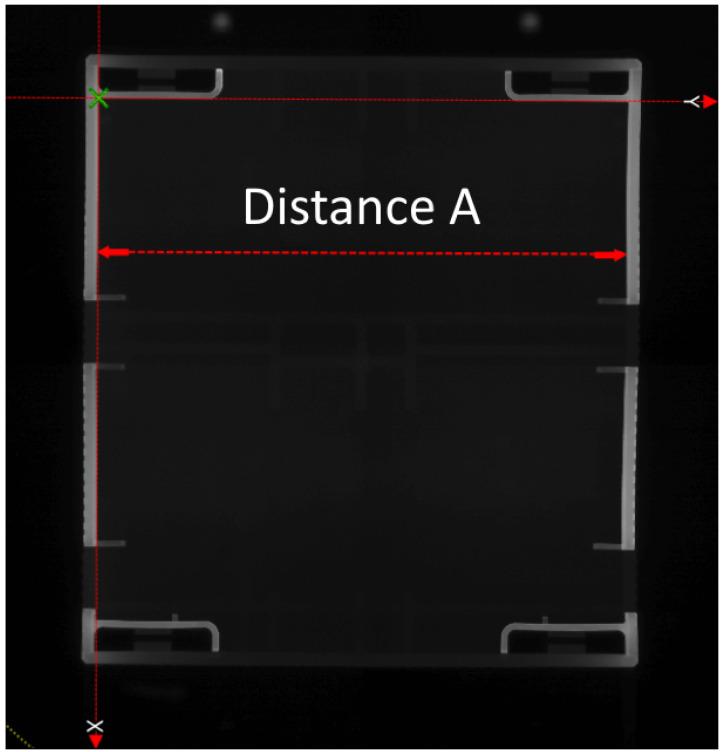
Geometrical measurement at X=65mm (top view).

In Figure 2, the quality properties resulting from the experiment are plotted over the cycles, using the measurement systems described in Section 2.1. The geometric dimension Distance A is shown, together with the part weight. Distance A was the inside diameter at the position indicated in Figure 3. The mean value of Distance A over all 1167 cycles was 84.9372 mm. Although the measured values of Distance A were obviously influenced by the disturbance variables, the variance was 0.00043 mm${}^{2}$. The parts weight reached a mean value of 58.92 g. Here, the variance is 0.0024 g${}^{2}$.

#### 2.2.3. Stacking Box Data Set I

**Figure 4:**
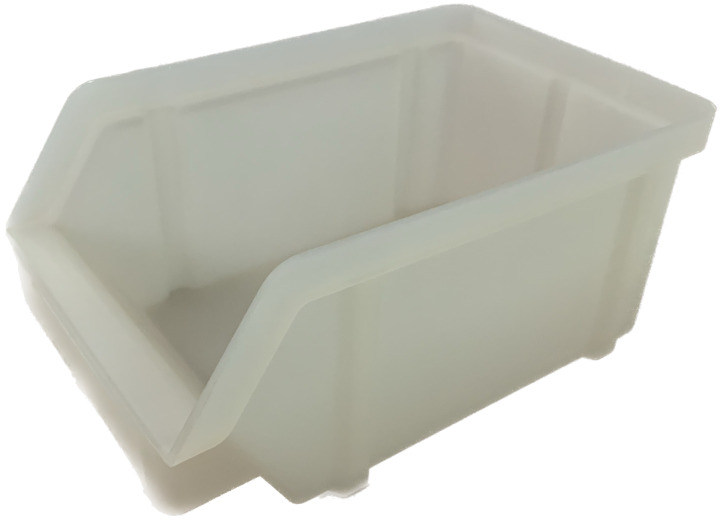
Stacking box.

The experiments for generating the Stacking Box Data Set I were carried out in the injection molding measuring cell, using a single-cavity mold with a hot runner for a molded part, in the form of a stacking box (Figure 4). The stacking box had external dimensions of about 160 × 100 × 73 mm (length × width × height), and a part weight of about 113.5 g (PA6-GF30). A polyamide (PA) granulate PA6-GF30 (Ultramid® B3EG6, manufactured by BASF SE, 67056 Ludwigshafen, Germany) was processed. The stacking box was a predominantly thick-walled (2 mm) injection-molded part with two partially freestanding side walls, which could be affected by warpage.

**Figure 5:**
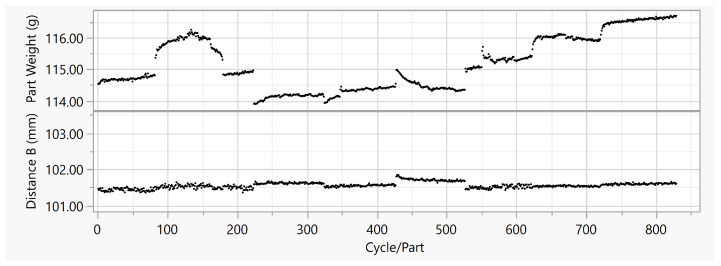
Part weight, Distance B vs. cycle or part in Stacking Box Data Set I.

The process conditions were changed in several trials (Table 2), by varying the moisture content of the PA granulate, and the mold temperature. The moisture content was varied on six levels (0.05 –0.18% ). The mold temperature was varied on three levels (70 °C, 80 °C, and 90 °C). The data contained both start-up processes and stable running processes, performed on three different days. The process was influenced both by controlled input parameters (mold temperature) and by disturbances (material moisture content), leading to a large variation in the molded part quality (see Figure 5). In order to create artificial disturbances, the moisture content was varied by prior treatment of the granulate. These values were only recorded to show the variance in the process input, and will not be used as model features.

**Figure 6:**
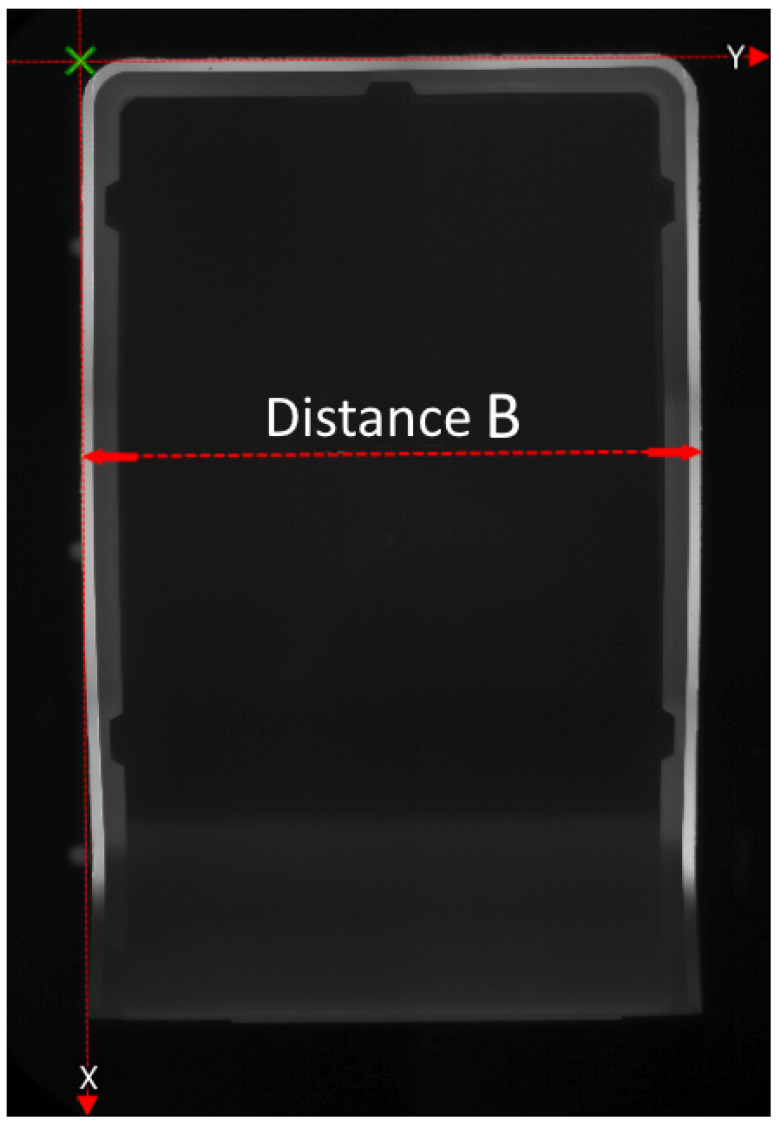
Geometrical measurement at X=25mm (top view).

Examination of the resulting measured quality data (see Figure 5) showed the clear influence of the input parameters and the disturbance variables induced in the experimental plan. The part weight reached an average of 115.16 g, and had a variance of 0.7084 g${}^{2}$ over all 829 cycles. The geometric dimension Distance B (see Figure 6), an outside diameter, had a mean value of 101.55 mm and a variance of 0.0068 mm${}^{2}$.

#### 2.2.4. Stacking Box Data Set II

The experiments for the Stacking Box Data Set II were also performed with the mold for the stacking box. A polyamide granulate PA6-GF30 (Repol Dinalon® B1S25 G30-0288) was used. The material was dried according to the manufacturer’s specifications. As in the other experiments, at the beginning of each test day, a few cycles were run to represent the machine start-up in the data, before the process was artificially influenced (Table 3). Next, the injection flow and holding pressure were decreased. To simulate a typical process interruption, the machine was paused for 15 min, with the melt staying in the barrel with switched-on barrel heaters, before starting the next batch of cycles. Finally, the barrel and hot runner temperatures were varied.

**Figure 7:**
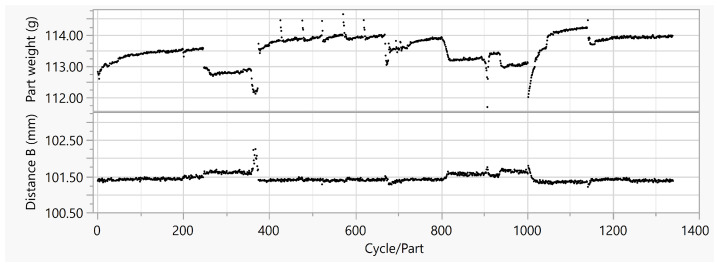
Part weight, Distance B vs. cycle or part in Stacking Box Data Set II.

As shown in Figure 7, the observation of the measured quality properties over 1332 cycles again shows the clear influence of the experimental design. The part weight reached a mean value of 113.54 g, with a variance of 0.1929 g${}^{2}$. The measurement of the geometric quantity Distance B yielded a mean value of 101.44 mm, with a variance of 0.0116 mm${}^{2}$.

## 3. Machine Learning Methodology

Regression methods are used to predict numeric values (the target values) based on given features (explanatory variables). For molding quality prediction in injection molding, the process data are considered as features: the scalar features or the elements of the time series data. A measure of product quality data can be used as the target.

Regression models belong to the field of supervised machine learning: this means that a model has learnable parameters, which have to be determined through a training procedure that takes both features and target values for a representative set of instances. The learned model can then be used on data where the target values are unknown, to predict them. For the molding process, this means that, for a number of cycles, the desired quality feature has to be measured. Once the model is learned properly, it can be applied to new products, and can predict the quality feature with a certain precision: if the latter is sufficient, further expansive quality measurements can be omitted and replaced by the regression model’s predictions.

Next to their learnable parameters, many models also have to be parameterized by hyperparameters, which are variables that are chosen, rather than learned, by the user, before the learning process begins. Different choices yield different models, and are thus an influence on the resulting predictive power of a model. Usually, a fixed set of these hyperparameters is selected and, for each combination, a model is learned and evaluated. Then, the hyperparameter combination that yields the highest quality is chosen to be run in production.

To evaluate a model, again, feature data with known targets (in-cycle sensor measurements with known quality results) are used. The model is run on the features, and the thus-predicted value is compared to the actual target.

### 3.1. Nested Cross-Validation

The above-described learning process comprises three steps: learning the learnable parameters; selecting the best hyperparameters; and comparing different algorithms against one other. In machine learning, it is well known that for these three tasks, three different (disjoint) sets of data have to be used, to minimize the risk of overfitting the model [16]. The latter means that the model might pick up on patterns, in the data that it is trained on, that are specific artifacts of that data, but that are not generally true: in such cases, the model would yield good predictions on the data that it is trained on, but severely worse predictions on previously unseen data. Thus, the training performance overestimates the actual predictive power in the real-use case. As observed in Section 3, due to the large number of individual values per cycle or iteration, the number of features is very high (compared to the number of elements—cycles—in the data sets), and the risk of overfitting is particularly high.

To counter these effects, we employed the procedure of repeated, nested cross- validation [16], a procedure which splits the available data (features with targets), at random, into three parts: the training data, for training learnable parameters; the validation set, for selecting the best hyperparameters; and the test data, for evaluating the best-configured model, and for comparing it to other algorithms. Thus, each part has its own subset of the data, and the models are evaluated on data that has neither been used for training nor for hyperparameter optimization (and so the evaluation is similar to the real-use case, where the model also encounters new, unseen data). To avoid random artifacts resulting from choosing one particular split, algorithms are compared on multiple splits.

In our experiments, we repeated each cross-validation five times, and split the data into 10 folds: each time, one of the splits was selected as test data; the remaining nine splits were merged, and again split into a 10-fold cross-validation (hence, nested cross-validation), to compare the hyperparameters and train the models. Thus, for each algorithm and each selected set of features, 50 tests were conducted, using models that had each been optimized on 50 data sets, yielding a total of 2500 experiments per algorithm and feature set.

Consequently, each considered algorithm was evaluated on 50 different subsets of the available data. By the design of the procedure, each instance in a data set was used five times in a test set: thus, every cycle in the data had the same influence on the overall results. The numbers reported in the next section were averaged over these 50 runs.

### 3.2. Feature Selection

The feature categories available for each molding cycle were scalar data and time series data. To determine the influence of the selection of the feature data category on prediction quality, three feature combinations were evaluated separately, and were then compared: the models were computed, based only on the scalar data (s), only on the time series (t), and on the combination of both data categories (st). While the scalar data for a cycle consisted of only 12 individual values, the time series consisted of a total of 4098 values. In the models, where the combination of both data categories, i.e., scalar and time series data, was used as a feature, we had a total feature count of 4110 values per cycle.

### 3.3. Targets

Machine learning models have to be optimized, with respect to exactly one quantity; therefore, in our experiments, a single quality property was used, to learn and compare models. We conducted two series of experiments: one where the target was the weight of the corresponding molded part, and one where a particular geometric dimension of the part served as the target.

### 3.4. Evaluation Measures and Significance

In the comparison of different models, as well as in the comparison of different hyperparametrizations, evaluation measures were needed, that summarized the difference between the actual target and the predicted value over multiple cases into one score. For the optimization of hyperparameters, and during the training process, the mean squared error (MSE) was used, i.e.,

(1) $$\mathrm{MSE}=\frac{1}{n}\sum_{i=0}^{n}{\left(y_{\mathrm{ref},i}-y_{\mathrm{pred},i}\right)}^{2}$$

where *N* is the number of instances (cycles) in the data set, $y_{\mathrm{ref},i}$ is the actual value (the measured quality of a cycle’s product), and $y_{\mathrm{pred},i}$ represents the corresponding predicted values.

In the overall comparison, we used two different measures. The coefficient of determination

(2) $$R^{2}=1-\frac{\sum_{i=0}^{n}{\left(y_{\mathrm{ref},i}-y_{\mathrm{pred},i}\right)}^{2}}{\sum_{i=0}^{n}{\left(y_{\mathrm{ref},i}-y_{\mathrm{mean}}\right)}^{2}}$$

was used to quantify the explained variance of the data: to that end, it related the mean squared error of the predictions (numerator) to the variance of the data (denominator). Higher values meant better predictions.

The mean absolute percentage error (MAPE)

(3) $$\mathrm{MAPE}=\frac{100}{n}\sum_{i=1}^{n}\left|\frac{y_{\mathrm{ref},i}-y_{\mathrm{pred},i}}{y_{\mathrm{ref},i}}\right|$$

quantified the average relative error, i.e., the prediction error, relative to the actually expected value.

$R^{2}$ was directly dependent on MSE, and therefore punished large differences more than smaller ones. In contrast, MAPE focused on the average relative deviation from the expected value, and was more easily interpretable, as it directly stated by how many percentage points the predictions were off, on average.

When we compared the results of two algorithms, or the same algorithm on two different feature sets, we compared their average performance on 50 different subsets of the data (see above). While the win of one algorithm over another on only one data set could be the result of random artifacts, this became less likely when compared to multiple data sets (here, 50). To quantify this, we followed the suggestion in [17], and used the Wilcoxon signed-rank test [18] to confirm whether the observed differences between two models were significant.

### 3.5. Baselines

When approaching a prediction problem, it is not a priori clear how hard this problem will be—are predictions easy or difficult? To get an impression of the difficulty of a problem, and to get a grasp of the value of investing in complex regression models, it helps to compare them to the results of simple baselines.

In regression, two baselines are common: baseline mean and baseline median, which can be seen as dummy models or naive predictors. Baseline mean simply predicts the mean target of the values seen during training, whereas baseline median does the same with the median of the training targets. Both baselines completely ignore the actual feature data, and always “predict” the same constant value (the mean or median, respectively).

Every regression model that is considered for use in production should significantly outperform both baselines: only a significantly higher prediction quality justifies the effort of training and employing such models. If baseline mean or median models provide high coefficients of determination $R^{2}$, and low errors (MAPE), this indicates targets with low variance, and thus, possibly, tasks where prediction models are inappropriate [19].

### 3.6. Regression Algorithms

In order to investigate the influence of the selection of available features (scalar, time series, scalar, and time series) on the prediction performance of the molded part quality prognosis, five different state-of-the-art and well-established supervised machine learning regression algorithms were chosen. These model approaches were considered to be well-investigated, and were therefore suitable for benchmarking performance on different feature sets.

Robust implementations were available in the open-source machine learning software library, Scikit-learn, for the Python programming language. The following five algorithms were used:

- Decision Tree Regression [20], which learns a partition of the feature space by cutting through orthogonally to one of the feature axes. For each resulting segment of the space, one target value is learned, which is predicted for all instances that fall into that segment.
- *k* Nearest Neighbors [21], which classifies a new instance by identifying the *k* most similar instances from the training data, and computing their average target value as a prediction for the instance at hand.
- Linear Regression [22], which learns an affine–linear multivariate function, mapping the vector of features onto a real number (the predicted target).
- Ridge Regression [23], which is similar to Linear Regression, and adds a regularization component to the optimization in the learning process, that is designed to minimize overfitting.
- Support Vector Regression with radial basis function kernel (SVR RBF) [24], which also learns a linear model; however, the feature space is first transformed by a non-linear function—the RBF kernel. The resulting regression model is linear in the transformed space but non-linear in the original space of features.

All these algorithms have different hyperparameters, e.g. the number *k* of considered nearest neighbors in *k* Nearest Neighbors, or the heuristic by which a decision tree decides the next cut in the features space partition. Others include various numerical parameters controlling the influence of certain components in algorithms, such as the influence of the regularization in Ridge Regression, or coefficients in the RBF kernel. These hyperparameters have to be selected by the user. To find proper choices, a set of candidates is chosen and evaluated. The best-performing combination is then used in production (see Section 3.1).

Finally, the two baselines (mean and median) are also regression algorithms, albeit simple ones without hyperparameters.

## 4. Results

In this section, we present the results of our part quality regression experiments on all three data sets. In particular, we not only compared multiple typical regression algorithms, regarding their predictive power, but we also leveraged different types of features (s, st, or t), and compared the respective regression quality (using MAPE and $R^{2}$); therefore, we compared results using solely scalar features (s) to those using time series (t) or the combination of time series and scalar features (st).

All the reported values are averaged results from 50 experiments (10-fold cross-validation, repeated with five different splits). We tested the significance of the differences between those results (“s vs. t” or “s vs. st”): to that end, we followed the suggestion in [17], and used the Wilcoxon signed-rank test [18]. While most differences were confirmed significant, there were some where both types of features yielded comparable results: these cases are reported in italics. Naturally, this always includes the two baselines (mean and median), as they yielded the exact same results, independent of the chosen features. To estimate the variation of prediction performance within the 50 experiments, for each algorithm and feature combination, the standard deviations for R² and MAPE are given in Appendix A.

### 4.1. Housing Part Data Set

The results for the prediction of a parts weight in the Housing Part data set are shown in Table 4. The highest R² were obtained when using k Nearest Neighbors regression. Based on scalar features only, (**s**), $R^{2}$ was 0.660; with time series features only, (**t**) $R^{2}$ was 0.750. The highest R² of 0.777 was achieved with the combination of scalar and time series features (**st**). The lowest mean average percentage errors (MAPE) were also achieved by using *k* Nearest Neighbors regression—0.029% for (**s**), 0.024% (**t**), and 0.023% for (**st**): thus, the best value was reached using **st**. The $R^{2}$ for the baselines mean and median were all below zero; however, the MAPEs were not particularly high, at 0.063% and 0.062% , respectively. The results for Target Distance A in Table 5 show the highest $R^{2}$ results for the Ridge Regression. The highest $R^{2}$ was reached with 0.502, and with the lowest MAPE of 0.017% with the **st** features. As with molding weight, the $R^{2}$s for the two baseline comparisons were negative, although very low MAPEs were also obtained here.

### 4.2. Stacking Box Data Set I

The results of the model comparison on the Stacking Box Data Set I are shown in Table 6. Compared to the results on the previous data set, the $R^{2}$s of all the models were much higher. Except for the Decision Tree Regression, the highest $R^{2}$s were obtained for the st features. The $R^{2}$s were 0.993 for SRV RBF, 0.993 for K Nearest Neighbors, 0.992 for Ridge Regression, and 0.989 for Linear Regression—very close to each other. The MAPEs were similar: they were also very close to each other at a low level. The results of the baseline mean and median comparison show the MAPEs that were significantly higher than those of the regression models. Table 7 shows the results for the model comparison for the target geometric dimension, Distance B: here, the SVR RBF performed best, with an $R^{2}$ 0.785 for the st features. The MAPE for the SVR RBF was 0.027% , about half of the baseline mean and median MAPEs.

#### Stacking Box Data Set II

The results for the Stacking Box Data Set II, in Table 8—also performed with the stacking box tool—show similar high R² for part weight, compared to the Stacking Box Data Set I. Decision Tree Regression, K Nearest Neighbors, Linear Regression, Ridge Regression, and SVR RBF all had an $R^{2}$ above 0.900 for the st and t features. The highest $R^{2}$ of 0.991 was achieved with Ridge Regression and the st features: with a low MAPE of 0.023, it was much more accurate then the baseline models. The lowest errors were achieved across all models with the st features.

The results for Distance B for the Stacking Box Data Set II in Table 9 have the highest $R^{2}$s for any geometric target in this work. The st and t features achieved the highest $R^{2}$s. The MAPEs behaved analogously: they were also below those of the baseline models.

## 5. Discussion

### 5.1. Housing Part Data Set

The best performing model for predicting the part weight in the Housing Part data set was obtained with the *k* Nearest Neighbors algorithm, and achieved the highest $R^{2}$, of 0.777, and the lowest MAPE, of 0.023% , with the feature combination of scalar data and time series (st). For part weight prediction, the $R^{2}$ was low: this was due to the low variance of the measured part weights in the data set. The low variance was also reflected in the comparatively low MAPE of 0.062% of the baseline median algorithm. Despite the large manual variation of the input parameters when performing the experiments, the resulting variance in the part quality was not large enough: the machine-mold combination had yielded a very robust process. While this is good news for the operators, it makes it hard to learn the influences of process parameters; nevertheless, the *k* Nearest Neighbors algorithm, with its MAPE of 0.023% , provided significantly better predictions than the baselines. The stability of the predictive performance across all test data set splits was also reflected in the low standard deviations for MAPE (shown in Table A1). For the prediction of the geometric dimension, lower prediction performances were achieved across all algorithms. Although this tendency can also be observed in other studies [9], the particularly low level of $R^{2}$ was again due to the low variance of the target in the data set (see baseline MAPE).

In summary, for this data set, it can be deduced that the best prediction results were achieved based on the combination of time series and scalar data. As the second-best results were achieved with the time series alone, it can be concluded that the time series were highly relevant.

### 5.2. Stacking Box Data Set I

For the Stacking Box Data Set I, significantly higher $R^{2}$s were achieved for the prediction of the target part weight: this was due to the significantly higher variance of the measured target data, and was also represented by the high MAPE of the baseline. The highest $R^{2}$ for the individual algorithms were above 0.980: except for one outlier (Decision Tree Regression), these were achieved by the feature combination of time series and scalar data. The algorithm best suited for this prediction task was, again, the K Nearest Neighbors algorithm, with an $R^{2}$ of 0.993 and a MAPE of 0.036% . The low standard deviations for $R^{2}$, of 0.0027, and MAPE, of 0.00050% , across all test data set splits, confirm the stable prediction performance of the K Nearest Neighbors algorithm (see Table A3). The SVM RBF achieved the same $R^{2}$, but had a higher MAPE, of 0.047% . For the prediction of the feature geometric dimension, again—due to lower variance and the baseline MAPE being one power of ten lower—a lower overall level of $R^{2}$ was achieved. The SVR RBF achieved the highest $R^{2}$, of 0.785, with a MAPE of 0.027% based on the combination of time series and scalar data. The same low MAPE was achieved by the K Nearest Neighbors algorithm, but with slightly lower $R^{2}$, of 0.780. Looking at the standard deviations in Table A4, it can be seen that the SVR-RBF algorithm achieved more stable predictions in this case.

Similar to the Housing Part data set, it can be summarized for this data set that the best prediction performance was achieved by a combination of time series and scalar data as features.

### 5.3. Stacking Box Data Set II

Although the induced disturbances in the experimental design for the Stacking Box Data Set II were completely different compared to I, similar high R² for the prediction of part weight were achieved. The higher $R^{2}$ for the feature combination of time series and scalar data across all algorithms were also clear for this data set. The results of the analysis for the geometrical target with slightly higher variance and baseline MAPE, show the highest $R^{2}$ for geometrical targets in this investigation: they were achieved with the feature combination. For *k* Nearest Neighbors, even the features time series alone showed a slightly higher $R^{2}$, but the MAPE were the same.

### 5.4. Further Discussion

In the presented comparison, the best prediction models, with the highest coefficients of determination across all algorithms, were archived for the feature combination of time series and scalar data for the Target Weight in the Stacking Box I and II data sets. In addition to the low MAPE for these algorithms, the standard deviations of the MAPE were also very low, which indicated stable learning processes and reliable prediction performance results. For the Target Distance B, the highest coefficients of determination across all the algorithms were lower. The relatively large differences in prediction performance between the two quality attributes can be explained by the lower variance in the measured quality data of the Distance B target; however, the MAPEs of the best models for Target Distance B in the Stacking Box I and II data sets were still very low and, depending on the manufacturing tolerances, did not exclude an application for process monitoring in practice. The same observation can be made for the Housing Part data set: here, the measured quality data varied even less, and did not allow for better coefficients of determination for both target categories.

To generate well-performing prediction models, it is important to use representative training data: we saw this tendency clearly when comparing the results from the Housing Part data set to those from the Stacking Box I and II data sets. It can be assumed that the lower the variance of the targets (with still-varying feature data), the more difficult the modeling task is for the algorithms. It can be concluded that the nature of the data sets, especially the variance of the measured quality data, has a strong influence on the prediction performance characteristics. It is important to test new approaches on different large data sets with realistic process influences. In summary, however, it can be said that even if the prediction performance characteristics of the presented algorithms are low, the quality in the real process also varies little, i.e., in the context of process monitoring for injection molding, small deviations from product specifications are to be expected.

The experiments confirm once more that, by including time series features, the performance of ML prediction algorithms can be improved.

## 6. Conclusions

The evaluation of the extensive data sets shows that time series and the combination of time series and scalar data as features allow significantly higher coefficients of determination and lower errors, i.e. better prediction models.

The time series were used directly as features for the models, without reducing their information content through prior formation of indices. The results show that the inclusion of injection pressure curve and injection flow curve, as features for molding quality prediction in injection molding, produces a significant improvement in prediction quality: therefore, high-resolution time series should be considered directly as features in future process monitoring methods based on the prediction of molding quality models.

The time series contain more information about the process dynamics (e.g., effect of disturbances) than do aggregated scalar values. In our experiments, we demonstrated that standard ML algorithms are able to utilize this additional information for the benefit of the resulting prediction accuracy. Moreover, we can be confident that the increase in accuracy from including time series is a general tendency, as it showed repeatedly in different settings (different parts, quality measures, and process variations).

The price for the higher prediction accuracy is the inclusion of significantly more features: in our example, the feature count went up from 12 scalar features to 4089 different features. A large number of features means high complexity and many degrees of freedom in the learning process of machine learning algorithms: this, in turn, is known to cause these algorithms to overfit. It is therefore imperative to evaluate such settings carefully: in this work, we met this challenge by training with extensive experimental data and state-of-the-art validation methods, applying nested cross-validation followed by significance tests. We saw stable results in all comparisons, with either comparable results or (in most cases) significant improvements when including the full time series.

Furthermore, we demonstrated that a comparably high prediction performance can be achieved without sensor data from the mold: this suggests that the relevant information required for the models is already contained in the injection pressure and injection flow curves from the machine’s internal sensors. This would enable the use of process data-based quality prediction models in practice, for injection molding processes without in-mold sensors. Of course, the mandatory effort for creating a training data set (collecting quality data) for each new injection molding process is not reduced; however, when using prediction algorithms, such measurements have to be taken only for a set of training instances, instead of every produced item.

In future research, the presented approach will be extended by applying machine learning methods that can be specifically adapted for the interpretation of time series as features using Convolutional Neural Networks (CNN): these may be even better suited to extracting relevant information from the data. CNN can be used to make the information contained in the dynamic context of the individual values within a time series accessible for the model. The data sets generated and the results presented in this paper will serve as the basis for this future work. The data sets will be made publicly available with the publication of this paper, and can be used by the scientific community for comparison.

## Figures and Tables

**Table 1:** Varied parameters for Housing Part data set.

Cycles	Day	Machine State	Barrel and Hot Runner Temperatures	Injection Flow	Pausing Time	Mold Temperatures
0001–0100	1	start-up				
0101–0166	1	running	+5%			
0167–0178	2	start-up				
0179–0253	2	running	−5%			
0254–0323	2	running	−10%			
0324–0384	2	running		−10%		
0385–0467	2	running		+10%		
0458–0528	2	running		+20%		
0529–0589	3	start-up				
0590–0651	3	running			15 min	
0652–0712	3	running			35 min	
0713–0773	3	running			15 min	
0774–0834	3	running			15 min	
0835–0893	4	start-up				
0894–0983	4	running				+20 ${}^{\circ }$C
0984–1042	4	running				+30 ${}^{\circ }$C
1043–1174	4	running				+10 ${}^{\circ }$C

**Table 2:** Varied parameters for Stacking Box Data Set I.

Cycles	Day	Machine State	Avg. Moisture Content	Mold Temperature
001–089	1	start-up	0.066%	90 ${}^{\circ }$C
090–187	1	running	0.097%	90 ${}^{\circ }$C
188–303	1	running	0.150%	90 ${}^{\circ }$C
304–379	2	start-up	0.086%	90 ${}^{\circ }$C
380–465	2	running	0.180%	90 ${}^{\circ }$C
466–526	2	running	0.046%	90 ${}^{\circ }$C
527–625	3	start-up	0.083%	80 ${}^{\circ }$C
626–728	3	running	0.067%	90 ${}^{\circ }$C
729–829	3	running	0.067%	70 ${}^{\circ }$C

**Table 3:** Varied parameters for Stacking Box II dataset.

Cycles	Day	Machine State	Injection Flow	Holding Pressure	Pausing Time	Barrel and Hot Runner Temperatures
0001–0199	1	start-up				
0200–0247	1	running	−10%			
0248–0374	1	running	−10%	−10%		
0375–0425	2	running				
0426–0476	2	paused			15 min	
0477–0522	2	paused			15 min	
0523–0571	2	paused			15 min	
0572–0619	2	paused			15 min	
0620–0669	2	paused			15 min	
0620–0705	3	start-up				
0706–0802	3	running				
0803–0900	3	running				−5%
0901–0907	3	running				−10%
0908–1002	3	running				−5%
1003–1043	4	start-up				
1044–1141	4	running				+ 5%
1142–1240	4	running				−10%
1241–1340	4	running				+ 10%

**Table 4:** Data Set Housing Part I: results for Target Weight. The differences in comparisons of st or t vs. s were tested for significance, using the Wilcoxon signed-rank test (α=0.05). Where the test did not confirm significant differences, the respective st or t values are printed in italic. The highest $R^{2}$ and the lowest MAPE are shown in bold for each algorithm.

	R²			MAPE in %		
	s	st	t	s	st	t
Decision Tree Regression	0.527	***0.553***	*0.542*	**0.031**	0.033	0.034
K Nearest Neighbors	0.660	**0.777**	0.750	0.029	**0.023**	0.024
Linear Regression	**0.308**	*−0.129*	*−0.187*	0.047	**0.037**	0.037
Ridge Regression	0.306	0.463	**0.506**	0.047	0.031	**0.030**
SVR RBF	**0.172**	0.066	0.060	**0.057**	0.062	0.062
Baseline mean	−0.010	*−0.010*	*−0.010*	0.063	*0.063*	*0.063*
Baseline median	−0.056	*−0.056*	*−0.056*	0.062	*0.062*	*0.062*

**Table 5:** Data Set Housing Part II: Results for Target Distance A. The highest $R^{2}$ and the lowest MAPE are shown in bold for each algorithm. The differences in comparisons of st or t vs. s are all significant according to the Wilcoxon signed-rank test (α=0.05).

	R²			MAPE in %		
	s	st	t	s	st	t
Decision Tree Regression	0.179	0.314	**0.349**	0.021	0.019	**0.019**
K Nearest Neighbors	0.082	0.424	**0.456**	0.022	0.018	**0.018**
Linear Regression	**0.222**	−0.383	−0.447	**0.021**	0.025	0.025
Ridge Regression	0.223	**0.502**	0.494	0.021	**0.017**	0.017
SVR RBF	0.209	**0.461**	0.449	0.021	0.018	**0.018**
Baseline Mean	−0.010	−0.010	−0.010	0.023	0.023	0.023
Baseline Median	−0.011	−0.011	−0.011	0.023	0.023	0.023

**Table 6:** Stacking Box Data Set I: results for Target Weight. The differences in comparisons of st or t vs. s were tested for significance, using the Wilcoxon signed-rank test (α=0.05). Where the test did not confirm significant differences, the respective st or t values are printed in italic. The highest $R^{2}$ and the lowest MAPE are shown in bold for each algorithm.

	R²			MAPE in %		
	s	st	t	s	st	t
Decision Tree Regression	**0.985**	0.980	0.899	**0.044**	0.056	0.134
K Nearest Neighbors	0.980	**0.993**	0.946	0.056	**0.036**	0.094
Linear Regression	0.932	**0.989**	0.877	0.147	**0.055**	0.192
Ridge Regression	0.932	**0.992**	0.920	0.147	**0.047**	*0.149*
SVR RBF	0.987	**0.993**	0.943	0.060	**0.047**	0.115
Baseline Mean	−0.012	*−0.012*	*−0.012*	0.654	*0.654*	*0.654*
Baseline Median	−0.105	*−0.105*	*−0.105*	0.640	*0.640*	*0.640*

**Table 7:** Stacking Box Data Set I: results for Target Distance B. The differences in comparisons of st or t vs. s were tested for significance, using the Wilcoxon signed-rank test (α=0.05): where the test did not confirm significant differences, the respective st or t values are printed in italic. The highest $R^{2}$ and the lowest MAPE are shown in bold for each algorithm.

	R²			MAPE in %		
	s	st	t	s	st	t
Decision Tree Regression	**0.703**	*0.697*	0.557	0.033	**0.032**	0.037
K Nearest Neighbors	0.721	**0.780**	*0.711*	0.032	**0.027**	0.030
Linear Regression	0.039	0.524	**0.525**	0.063	0.042	**0.042**
Ridge Regression	0.039	**0.700**	0.697	0.063	**0.033**	0.033
SVR RBF	0.577	**0.785**	0.717	0.039	**0.027**	0.031
Baseline Mean	−0.013	−0.013	−0.013	0.063	0.063	0.063
Baseline Median	−0.021	−0.021	−0.021	0.063	0.063	0.063

**Table 8:** Stacking Box Data Set II: results for Target Weight. The differences in comparisons of st or t vs. s were tested for significance, using the Wilcoxon signed-rank test (α=0.05): where the test did not confirm significant differences, the respective st or t values are printed in italic. The highest $R^{2}$ and the lowest MAPE are shown in bold for each algorithm.

	R²			MAPE in %		
	s	st	t	s	st	t
Decision Tree Regression	0.870	**0.919**	0.908	0.060	**0.051**	0.053
K Nearest Neighbors	0.914	**0.972**	0.969	0.052	**0.030**	0.032
Linear Regression	0.738	**0.988**	0.985	0.132	**0.028**	0.030
Ridge Regression	0.739	**0.991**	0.988	0.131	**0.023**	0.026
SVR RBF	0.908	**0.973**	0.963	0.072	**0.046**	0.050
Baseline Mean	−0.010	*−0.010*	*−0.010*	0.323	*0.323*	*0.323*
Baseline Median	−0.050	*−0.050*	*−0.050*	0.323	*0.323*	*0.323*

**Table 9:** Stacking Box Data Set II: results for Target Distance B. The differences in comparisons of st or t vs. s were tested for significance, using the Wilcoxon signed-rank test (α=0.05): where the test did not confirm significant differences, the respective st or t values are printed in italic. The highest $R^{2}$ and the lowest MAPE are shown in bold for each algorithm.

	R²			MAPE in %		
	s	st	t	s	st	t
Decision Tree Regression	0.738	***0.788***	*0.783*	0.030	**0.026**	0.026
K Nearest Neighbors	0.829	0.880	**0.882**	0.027	0.023	**0.023**
Linear Regression	0.163	**0.743**	0.741	0.068	**0.035**	0.035
Ridge Regression	0.165	**0.870**	0.870	0.068	**0.025**	0.025
SVR RBF	0.609	0.837	**0.863**	0.037	0.025	**0.024**
Baseline Mean	−0.011	−0.011	−0.011	0.080	0.080	0.080
Baseline Median	−0.121	−0.121	−0.121	0.072	0.072	0.072

## Data Availability

Publicly available data sets were analyzed in this study. These data sets can be found here: https://github.com/sc4t1m/scatimdata.

## Appendix A

The following tables present the standard deviations (SD) for R² and MAPE for all data sets and targets. The standard deviation is an indicator of the stability of the prediction performance: each value is computed over the individual data splits in the context of the applied nested cross-validation (see Section 3.1).

**Table A1:** Housing Part data set: standard deviations for the results of Target Weight.

	SD for R²			SD for MAPE in %		
	s	st	t	s	st	t
Decision Tree Regression	0.1403	0.1345	0.1367	0.0043	0.0042	0.0043
K Nearest Neighbors	0.0769	0.0700	0.0795	0.0030	0.0029	0.0030
Linear Regression	0.0923	1.2863	1.3485	0.0038	0.0068	0.0069
Ridge Regression	0.0910	0.3270	0.2898	0.0038	0.0038	0.0034
SVR RBF	0.0709	0.0792	0.0617	0.0038	0.0036	0.0042
Baseline Mean	0.0126	0.0126	0.0126	0.0048	0.0048	0.0048
Baseline Median	0.0403	0.0403	0.0403	0.0053	0.0053	0.0053

**Table A2:** Housing Part data set: standard deviations for the results of Target Distance A.

	SD for R²			SD for MAPE in %		
	s	st	t	s	st	t
Decision Tree Regression	0.1587	0.1612	0.1599	0.0021	0.0018	0.0018
K Nearest Neighbors	0.1107	0.1290	0.1358	0.0023	0.0016	0.0015
Linear Regression	0.1785	0.6294	0.6715	0.0019	0.0028	0.0029
Ridge Regression	0.1796	0.1324	0.1363	0.0019	0.0013	0.0013
SVR RBF	0.1300	0.0973	0.0872	0.0022	0.0016	0.0016
Baseline Mean	0.0153	0.0153	0.0153	0.0025	0.0025	0.0025
Baseline Median	0.0132	0.0132	0.0132	0.0025	0.0025	0.0025

**Table A3:** Stacking Box Data Set I: standard deviations for the results of Target Weight.

	SD for R²			SD for MAPE in %		
	s	st	t	s	st	t
Decision Tree Regression	0.0100	0.0080	0.0396	0.0082	0.0087	0.0243
K Nearest Neighbors	0.0110	0.0027	0.0170	0.0079	0.0050	0.0129
Linear Regression	0.0112	0.0029	0.0314	0.0126	0.0050	0.0168
Ridge Regression	0.0111	0.0022	0.0186	0.0126	0.0043	0.0126
SVR RBF	0.0049	0.0015	0.0165	0.0057	0.0038	0.0122
Baseline Mean	0.0172	0.0172	0.0172	0.0333	0.0333	0.0333
Baseline Median	0.0658	0.0658	0.0658	0.0349	0.0349	0.0349

**Table A4:** Stacking Box Data Set I: standard deviations for the results of Target Distance B.

	SD for R²			SD for MAPE in %		
	s	st	t	s	st	t
Decision Tree Regression	0.0668	0.0767	0.1127	0.0026	0.0030	0.0044
K Nearest Neighbors	0.0483	0.0601	0.0770	0.0027	0.0027	0.0030
Linear Regression	0.0381	0.0990	0.1006	0.0050	0.0030	0.0031
Ridge Regression	0.0375	0.0642	0.0665	0.0050	0.0029	0.0029
SVR RBF	0.0606	0.0504	0.0751	0.0037	0.0024	0.0028
Baseline Mean	0.0130	0.0130	0.0130	0.0052	0.0052	0.0052
Baseline Median	0.0247	0.0247	0.0247	0.0053	0.0053	0.0053

**Table A5:** Stacking Box Data Set II: standard deviations for the results of Target Weight.

	SD for R²			SD for MAPE in %		
	s	st	t	s	st	t
Decision Tree Regression	0.0652	0.0504	0.0541	0.0131	0.0089	0.0102
K Nearest Neighbors	0.0419	0.0219	0.0214	0.0089	0.0060	0.0062
Linear Regression	0.0550	0.0048	0.0070	0.0128	0.0024	0.0023
Ridge Regression	0.0552	0.0052	0.0074	0.0130	0.0028	0.0029
SVR RBF	0.0308	0.0089	0.0231	0.0073	0.0039	0.0050
Baseline Mean	0.0168	0.0168	0.0168	0.0171	0.0171	0.0171
Baseline Median	0.0551	0.0551	0.0551	0.0187	0.0187	0.0187

**Table A6:** Stacking Box Data Set II: standard deviations for the results of Target Distance B.

	SD for R²			SD for MAPE in %		
	s	st	t	s	st	t
Decision Tree Regression	0.1709	0.1020	0.1077	0.0036	0.0034	0.0033
K Nearest Neighbors	0.0669	0.0486	0.0483	0.0030	0.0027	0.0028
Linear Regression	0.0881	0.1239	0.1233	0.0060	0.0031	0.0031
Ridge Regression	0.0861	0.0367	0.0366	0.0060	0.0025	0.0025
SVR RBF	0.1403	0.0587	0.0467	0.0075	0.0075	0.0075
Baseline Mean	0.0182	0.0182	0.0182	0.0058	0.0058	0.0058
Baseline Median	0.0586	0.0586	0.0586	0.0075	0.0075	0.0075
